# Development and Validation of a Prognostic Nomogram to Predict Cancer-Specific Survival in Adult Patients With Pineoblastoma

**DOI:** 10.3389/fonc.2020.01021

**Published:** 2020-07-24

**Authors:** Yajun Jing, Wenshuai Deng, Huawei Zhang, Yunxia Jiang, Zuoxiang Dong, Fan Fan, Peng Sun

**Affiliations:** ^1^Department of Neurosurgery, Affiliated Hospital of Qingdao University, Qingdao, China; ^2^Department of Pharmacology and Toxicology, University of Mississippi Medical Center, Jackson, MS, United States; ^3^Department of Nursing, Medical College of Qingdao University, Qingdao, China

**Keywords:** pineoblastoma, nomogram, prognosis, risk factor, C-index

## Abstract

Pineoblastoma (PB) is a rare neoplasm of the central nervous system. This analysis aimed to identify factors and establish a predictive model for the prognosis of adult patients with PB. Data for 213 adult patients with PB (Surveillance, Epidemiology, and End Results database) were randomly divided into primary and validation cohorts. A predictive model was established and optimized based on the Akaike Information Criterion and visualized by a nomogram. Its predictive performance (concordance index and receiver operating characteristic curve) and clinical utility (decision curve analyses) were evaluated. We internally and externally validated the model using calibration curves. Multivariate Cox regression analysis identified age, year of diagnosis, therapy, tumor size, and tumor extension as independent predictors of PB. The model exhibited great discriminative ability (concordance index of the nomogram: 0.802; 95% confidence interval: 0.78–0.83; area under the receiver operating characteristic curve: ranging from 0.7 to 0.8). Calibration plots (probability of survival) showed good consistency between the actual observation and the nomogram prediction in both cohorts, and the decision curve analyses demonstrated great clinical utility of the nomogram. The nomogram is a useful and practical tool for evaluating prognosis and determining appropriate therapy strategies.

## Introduction

The pineal gland is an endocrine gland in the midline of the brain that secretes melatonin to modulate circadian rhythms. Primary tumors arising from the pineal gland are termed pineal parenchymal tumors (PPTs). These tumors are rare, comprising <1% of all primary central nervous system neoplasms (1). Their occurrence is more commonly found in children [accounting for 3–8% (2) of all intracranial tumors] than in adults [accounting for 0.1–1.0% (3)]. Furthermore, the different histological types of PPTs exhibit varying growth patterns and histological features. According to the World Health Organization (WHO) classification of tumors of the central nervous system published in 2016 (4), PPTs are divided into four major subgroups: pineocytoma [WHO grade I, International Classification of Diseases for Oncology (ICD-O) 9361/1], PPT of intermediate differentiation (WHO grade II/III, ICD-O 9362/3), pineoblastoma (PB) (WHO grade IV, ICD-O 9362/3), and papillary tumor (WHO grade II/III, ICD-O 9395/3). PB, accounting for 25–50% of PPTs, is mostly observed in children, adolescents, and young adults (5–7). The average age of onset is 12.6 years, with a wide range (1–39 years) (8). However, there is a lower incidence of PB in adults vs. children.

Defined as a highly malignant embryonal tumor of the pineal gland, PB is a type of primary supratentorial middle primitive neuroectodermal tumor of the central nervous system (9). Similar to other malignant tumors, PB is traditionally linked to extremely poor prognosis with aggressive clinical behavior. PB often exhibits a high rate of relapse and propensity for seeding throughout the craniospinal axis (10, 11), as well as sporadic metastases to other parts of the body, such as the calvarial bones (12), vertebrae (13), lung (14), peritoneum (15), mandible (16), and pelvis (17). However, due to the rarity of PB, there is a lack of outcome data on patients with PB. At present, the bulk of information regarding overall survival in patients with PB available in the literature is in the form of case reports (18, 19) and single-institution studies comprising very small numbers of patients (5, 20). Although useful information was, to some extent, provided by these investigations, inadequate data and limitations, such as small sample studies, inherent biases, and insufficient statistical power, made it difficult to deduce reliable outcomes for patients with PB. Parikh et al. (21) concluded that pediatric and adult PB behave differently and should therefore be considered separately when analyzing response to surgical and adjuvant therapy. At present, prognostic factors for the survival of adult patients with PB are not clearly defined.

For the above reasons, we used the data of 213 adult patients with PB from the Surveillance, Epidemiology, and End Results (SEER) database to identify independent prognostic factors of adult patients with PB through Cox proportional hazards regression analysis. Moreover, we compared survival outcomes according to patient and tumor characteristics, as well as treatments received. Additionally, we attempted to establish a nomogram model based on the identified independent prognostic factors to provide a more accurate prediction of patient survival.

## Materials and Methods

### Data Resource

We acquired the data of patients with PB from the SEER database of the American National Cancer Institute using the latest SEER^*^Sat version 8.3.6 (https://seer.cancer.gov/), which contained the SEER 18 registries research data.

### Inclusion and Exclusion Criteria

**Figure 1:**
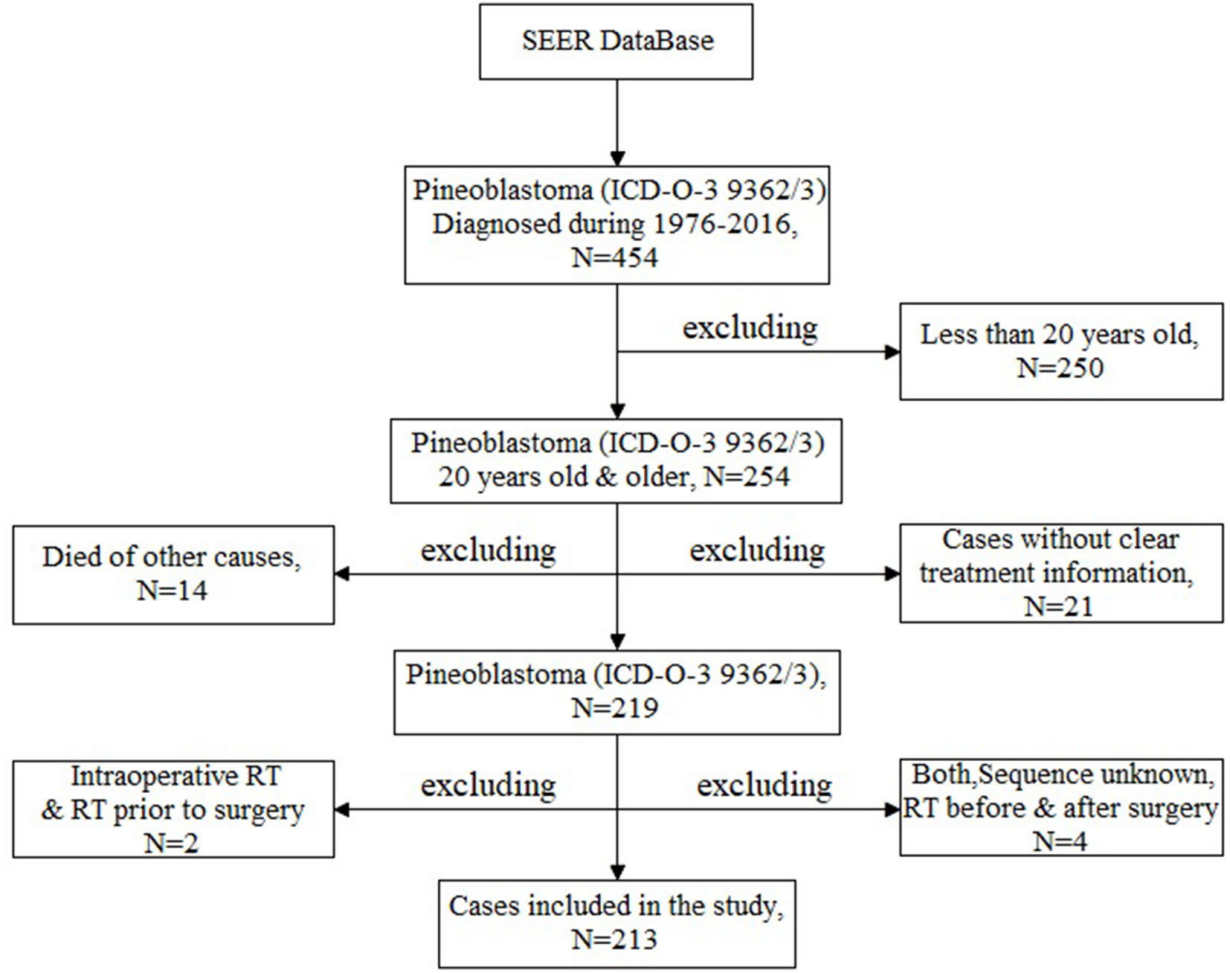
The flow chart of patient enrollment.

The aim of this study is to analyze the prognostic factors of adult patients with PB in a large sample dataset. In order to meet the research needs, inclusion and exclusion criteria were set up as follows. Inclusion criteria: (1)patients were diagnosed as PB between 1975 and 2016 [according to the ICD-O, Third Edition (ICD-O-3) issued by the WHO, ICD-O-3 code 9362/3]; (2) adult patients with PB who were older than 20 years old and younger than 80 at diagnosis; (3) cases with clear treatment information. Exclusion criteria: (1) other types of cancer were excluded according to the ICD-O-3 and Conversion of Neoplasms by Topography and Morphology from the ICD-O-2 to ICD-O-3 edited by the SEER Program National Cancer Institute; (2) patients aged <20 years; (3) patients without clear treatment information (unknown); (4) dead of other causes; (5) there was only one case in some treatment regimens (including the intraoperative beam radiotherapy [RT], RT prior to surgery, RT before and after surgery as well as sequence unknown, but both were given). Finally, 213 adult patients with PB were identified and selected for this analysis (Figure 1).

### Variables Selection

The following variables were included: age, sex, race, year of diagnosis, laterality (not including a paired site, right-origin of primary, left-origin of primary, and paired site, but no information concerning laterality), therapy [including RT; chemotherapy [CT]; surgical treatment; only biopsy; RT and CT, sequence unknown; RT after surgery; RT after surgery and CT, sequence unknown; and RT after non-primary surgical procedure and CT, sequence unknown], tumor extension (including invasive tumor confined to gland of origin, localized, not otherwise specified, adjacent connective tissue, adjacent organs/structures, and further contiguous extension; http://web2.facs.org/cstage0205/intracranialgland/IntracranialGland_bfy.html), and tumor size. The time from diagnosis to death or last follow-up was defined as survival time, and those cases lost to follow-up were censored by the SEER. Non-survival included death caused by PB. Furthermore, there were some missing data regarding variable tumor extension and size. Multiple imputation of missing data was performed using the R software (version 3.6.0; http://www.r-project.org/) to ensure a sufficient sample size and boost statistical power (22).

### Statistical Analysis

All cases were randomly divided into a primary cohort and a validation cohort at a ratio of 7:3 (23). The predictive model and nomogram were established using the data from the primary cohort; validation of the model was performed through the data of the validation cohort. The procedure for the construction of the predictive model and nomogram was as follows. First, a univariate Cox regression analysis was conducted to explore the possible prognostic risk factors; factors with a *p* < 0.1 in the univariate analysis were identified as possible risk factors. Second, all possible risk factors and those with a *p* < 0.05 in the multivariate analysis were identified as independent risk factors. Third, by selecting the factors with a *p* < 0.1 and given the limitation of the sample size in this study, we established the predictive model based on all risk factors and selected the optimal predictive model according to the results of the Akaike Information Criterion in a stepwise manner (24). Finally, the model was visualized by the nomogram.

The nomogram was validated internally in the primary cohort and externally in the validation cohort, as well as cross-validation in primary and validation cohorts by bootstrapping. The concordance index (C-index) and the time-dependent receiver operating characteristic (ROC) curve were applied to evaluate the accuracy and discriminative ability (25, 26). The association between the actual outcomes and the predicted probability was compared through calibration curves (27). Both discrimination and calibration were evaluated using bootstrapping with 1,000 resamples. Decision curve analysis (DCA) was employed to access the clinical utility of the nomogram (28).

The X-title software (version 3.61; Yale University, New Haven, CT, USA) was used to explore the best cutoff point of the continuous variables (including age, year of diagnosis, and tumor size), based on the log-rank test statistics (29). The optimal cutoff value was defined as the point reflecting the most significant split among the survival distributions of those factors with different categorical ages and tumor sizes. Lastly, the new categorical variables were generated based on the optimal cutoff point and used as predictors in the survival analysis.

All statistics analyses were performed using the R software (version 3.6.0; http://www.r-project.org/), X-title software (version 3.61; Yale University, New Haven, CT, USA), and SPSS (version 25.0; IBM Corporation, Armonk, NY, USA). A *p* < 0.1 was chosen as the criterion for excluding a variable from the multivariate Cox proportional hazards model, and a *p* < 0.05 denoted statistical significance for all other tests.

## Results

### Clinicopathological Characteristics of Patients

A total of 213 patients with PB were finally selected and analyzed in this study. The primary and validation cohorts consisted of 152 and 61 patients, respectively. Baseline information on the patients included in this study is shown in Table 1. Overall, the male-to-female ratio in this analysis was ~1:1. The majority of patients were white (*n* = 159, 74.6%) and aged 20–50 years (*n* = 154, 72.3%). In addition, a large proportion of the patients had laterality characteristics without a paired site (*n* = 207, 97%), and tumor extension characteristics confined to the gland of origin and localized, not otherwise specified (*n* = 135, 63.3%). About 80% of patients were diagnosed in the period from 2001 to 2016 (*n* = 169, 79.3%). Importantly, the ratio of cases with confirmed diagnosis by the positive histology were up to 97% (*n* = 207, 97%).

**Table 1:** Baseline clinicopathological characteristics and treatments of all patients.

**Variables**	**!! ± SD/N (%)**
	**Validation cohort Primary cohort**
**Age**	
20–44	38 (62.2%) 89 (58.6%)
≥45	23 (37.7%) 63 (41.4%)
**Sex**	
Male	32 (52.5%) 73 (48.0%)
Female	29 (47.5%) 79 (520%)
**Race**	
White	44 (72.1%) 115 (75.7%)
Others	17 (27.9%) 37 (24.3%)
**Year of diagnosis**	
1975–2000	8 (13.1%) 36 (23.7%)
2001–2016	53 (86.9%) 116 (76.3%)
**Tumor size**	29.67 ± 9.94 29 ± 11.49
**Laterality**	
Not a paired site	57 (93.4%) 150 (98.7%)
Paired site	4 (6.6%) 2 (1.3%)
**Therapy**	
Surgery	12 (19.7%) 28 (18.4%)
GTR	9 (14.8%) 20 (13.2%)
Subtotal resection	3 (4.9%) 8 (5.3%)
RT	10 (16.4%) 23 (15.1%)
Biopsy	0 (0.0%) 6 (3.9%)
CT	1 (1.6%) 2 (1.3%)
RT and CT, sequence unknown	5 (8.2%) 17 (11.2%)
RT after surgery	15 (24.6%) 15 (9.87%)
BRT after GTR	12 (19.67%) 1 (0.66%)
BRT after subtotal resection	3 (4.93%) 14 (9.2%)
RT after surgery and CT, sequence unknown	17 (27.9%) 23 (15.1%)
RT after GTR and CT	14 (23.0%) 17 (11.2%)
RT after subtotal resection and CT	3 (4.9%) 6 (3.9%)
RT after non-primary surgical procedure and CT, sequence unknown	1 (1.6%) 2 (1.3%)
**Extension**	
Confined to the gland of origin	34 (55.8%) 77 (50.7)
Localized, NOS	5 (8.2%) 19 (12.5%)
Adjacent connective tissues	6 (9.8%) 10 (6.6%)
Adjacent organs and structures	14 (23.0%) 44 (28.9%)
Further continuous extension	2 (3.2%) 2 (1.3%)
**Diagnostic confirmation**	
Positive histology	60 (98.4%) 147 (96.7%)
Radiography	0 (0.0%) 3 (2.0%)
Positive exfoliative cytology	1 (1.6%) 2 (1.3%)

*Paired site including right-origin of primary, left-origin of primary, and paired site, but no information concerning laterality. BRT, beam radiotherapy; CT, chemotherapy; GTR, gross total resection*.

### Univariate Analysis

**Figure 2:**
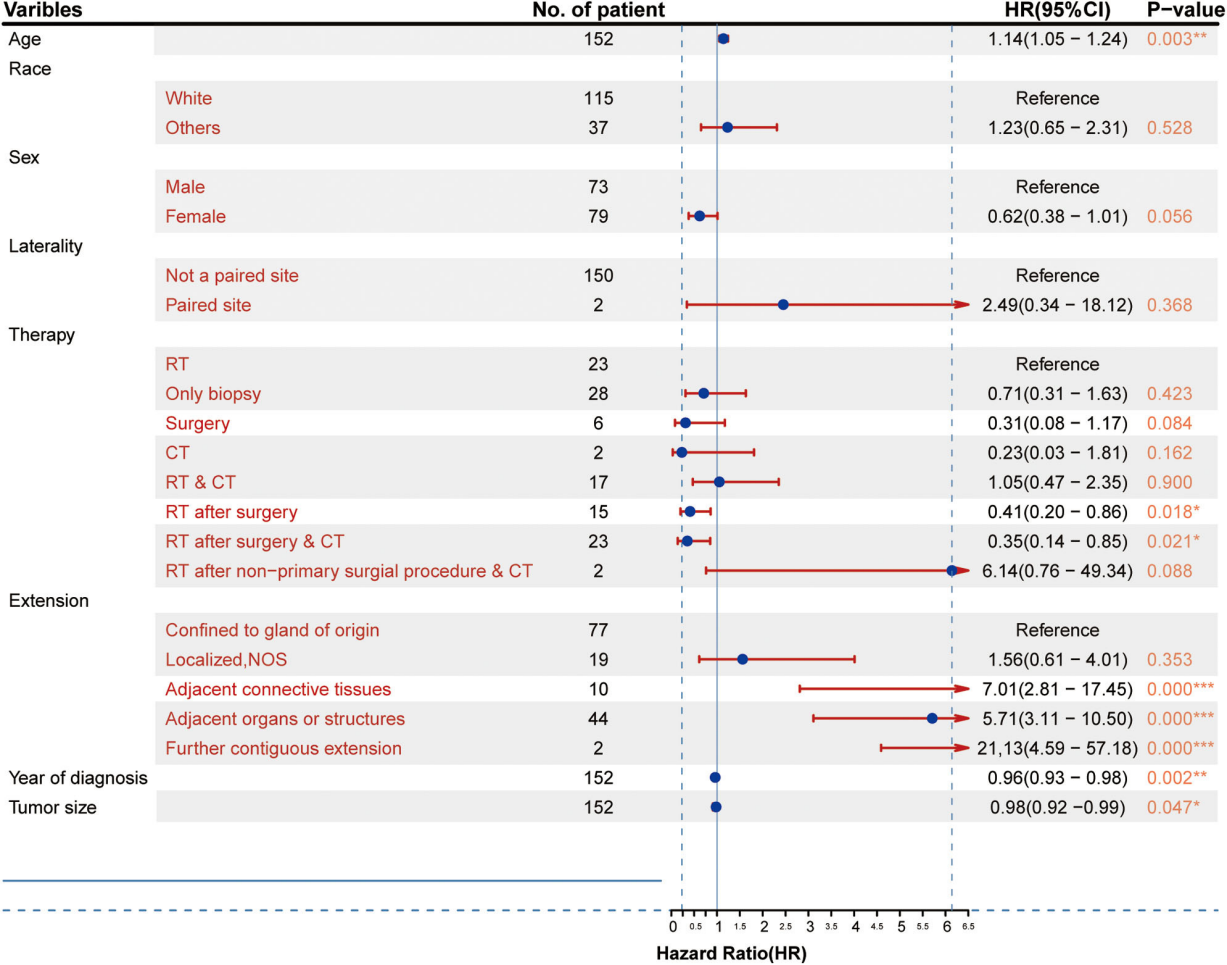
Clinicopathological characteristics of the patients and results of the univariate Cox proportional hazards analysis (HR, 95% confidence interval). *p < 0.05, **p < 0.01, ***p < 0.001.

As shown in Figure 2, the results of the univariate analysis revealed that age, year of diagnosis, therapy (RT after surgery and RT after surgery combined with CT), tumor size, and extent of tumor extension were significantly associated with patient survival. Other factors did not show statistically significant differences. However, taking into account the limitation of the sample size, those factors were not excluded from the multivariate analysis.

### Independent Prognostic Factors in the Primary Cohort

**Figure 3:**
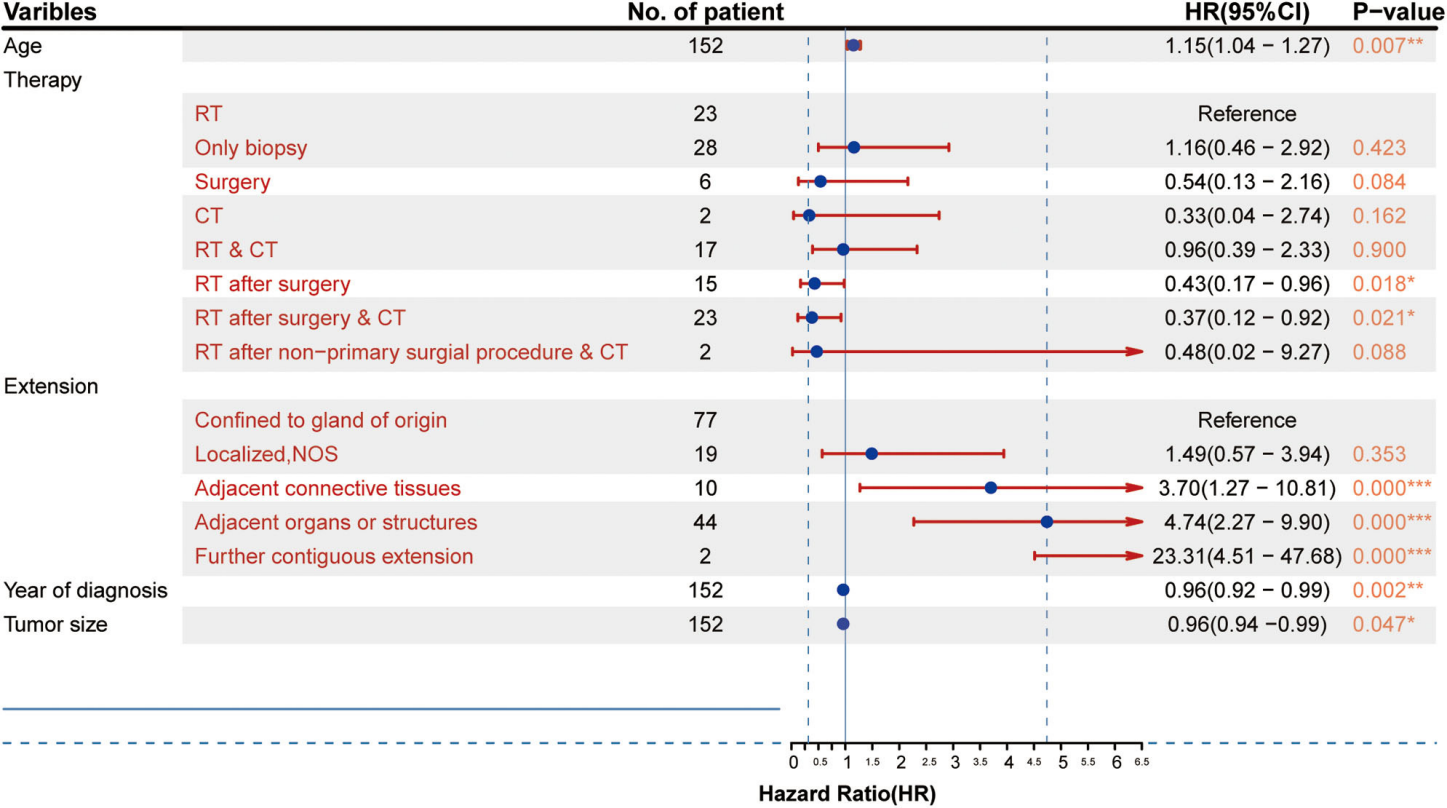
Results of the multivariate analysis of different factors (HR, 95% confidence interval). *p < 0.05, **p < 0.01, ***p < 0.001.

The results from the multivariate analysis are shown in Figure 3. The results demonstrated that the independent prognostic factors were age [hazard ratio (HR) = 1.15, *P* < 0.01], therapy strategies (RT after surgery: HR = 0.43, *P* < 0.05; RT after surgery combined with CT: HR = 0.38, *P* < 0.05), the scope of tumor extension (extension adjacent tissues: HR = 3.70, *P* < 0.05; extension adjacent organs and structures: HR = 4.74, *P* < 0.001; tumor further contiguous extension: HR = 23.31, *P* < 0.01), tumor size (HR = 0.96, *P* < 0.01), and year of diagnosis (HR = 0.96, *P* < 0.05). Sex (female: HR = 0.97, *P* > 0.1), race (others: HR = 1.14, *P* > 0.1), and laterality (paired site: HR = 5.44, *P* > 0.1) were not identified as independent prognostic factors.

**Figure 4:**
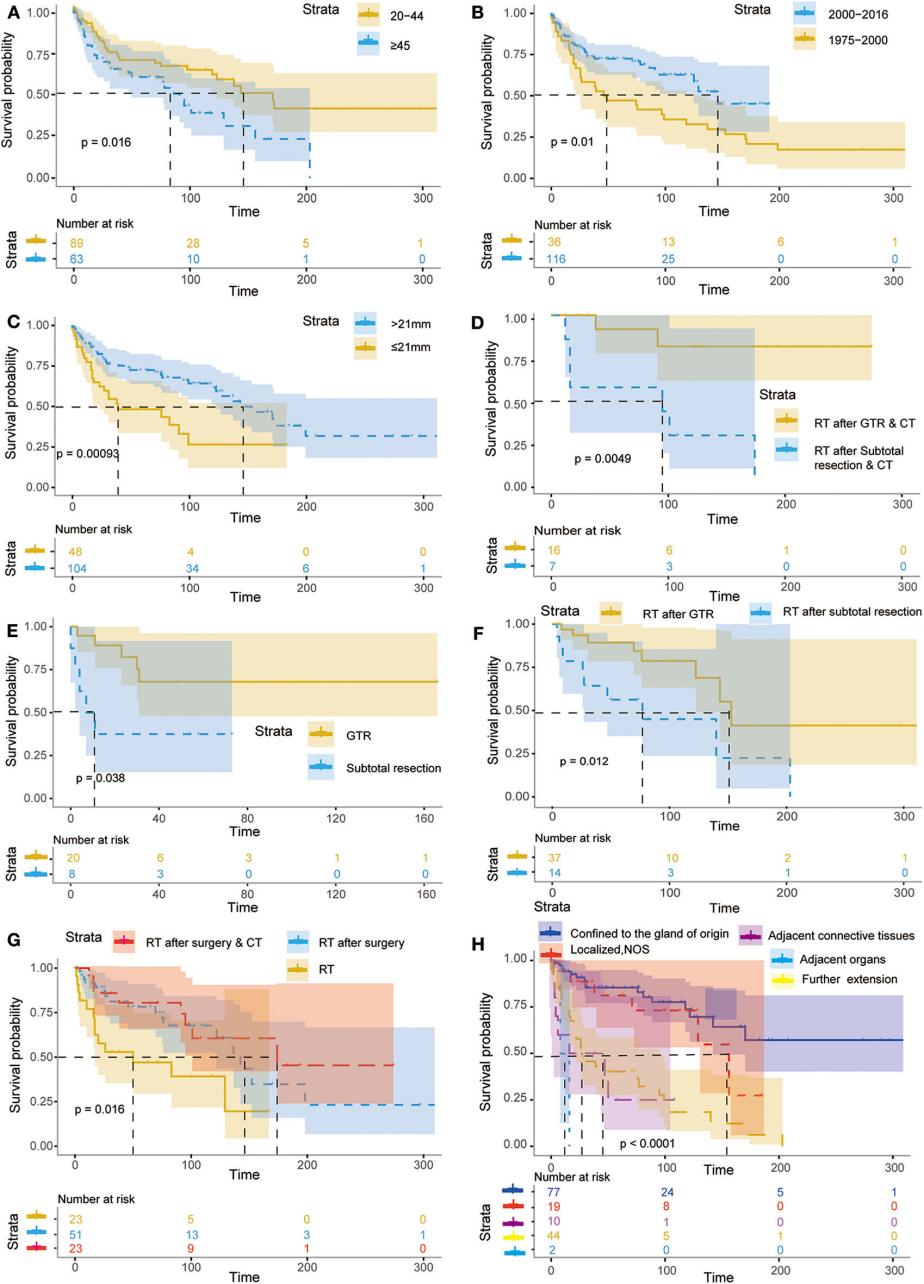
Kaplan–Meier curves for patients with PB according to different independent prognostic factors. The Kaplan–Meier curves for patients with primary PB according to age **(A)**, year of diagnosis **(B)**, tumor size **(C)**, RT after GTR & CT or RT after subtotal resection & CT **(D)**, GTR and subtotal resection **(E)**, RT after GTR or subtotal resection **(F)**, RT, RT after surgery & RT after surgery & CT **(G)**, extent of tumor extension **(H)**.

In addition, the effects of all dependent prognostic factors on survival of patients were assessed by survival analysis and shown by the Kaplan–Meier curves (Figure 4).

### Prognostic Nomogram of Overall Survival

**Figure 5:**
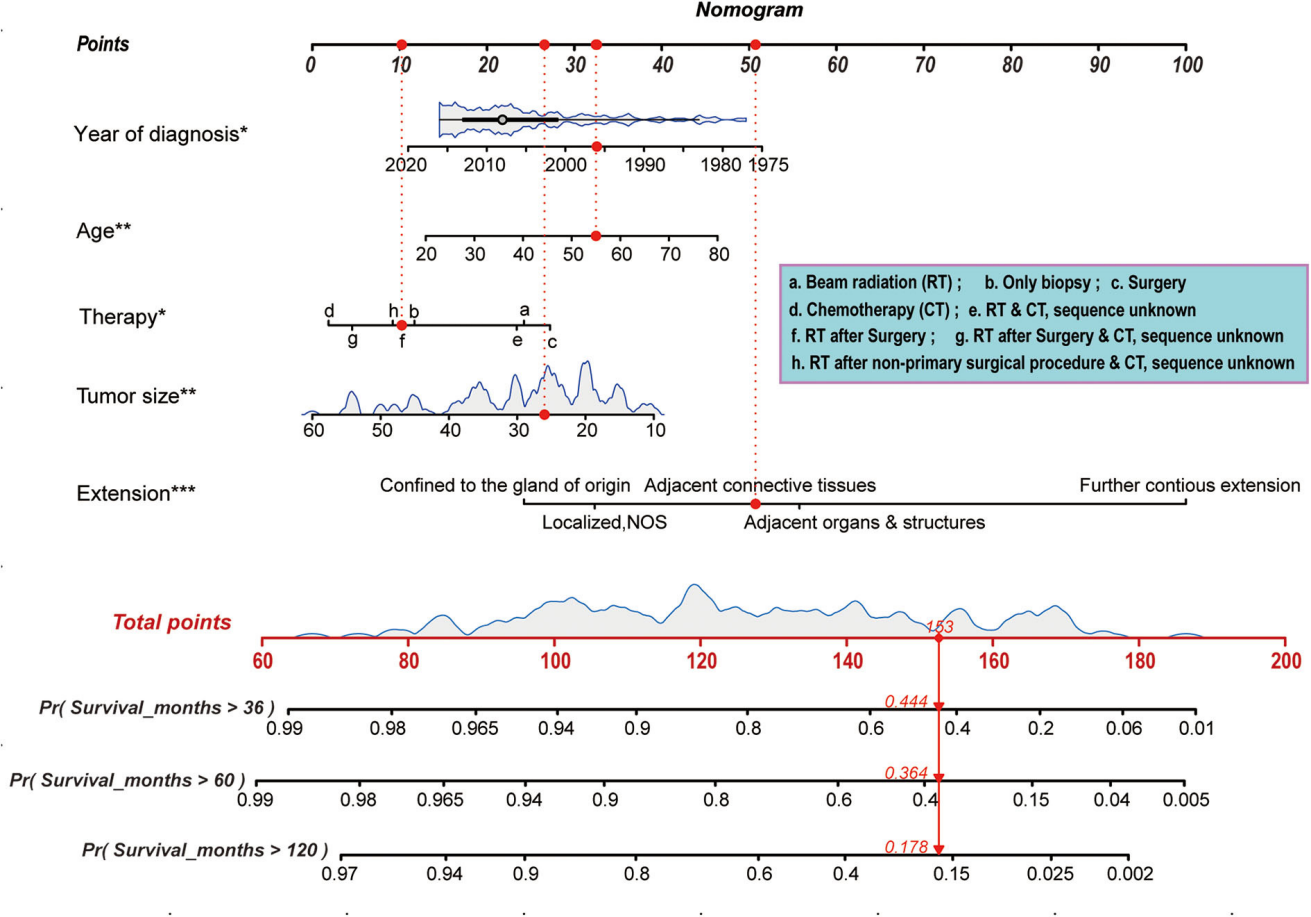
Nomogram predicting 36-, 60-, and 120-month cancer-specific survival for patients with PB. Prognostic factors including age, race, tumor extension, tumor size, and therapy, and the scores assigned on the points scale could match each level of every variable on the nomogram. Thus, a total score was obtained by adding the score from various variables or their levels. Finally, the 36-, 60-, and 120-month cancer-specific survival for each individual patient could be estimated on the basis of the total score. *p < 0.05, **p < 0.01, ***p < 0.001.

The prognostic nomogram, shown in Figure 5, was constructed based on the optimization results of the Akaike Information Criterion protocol in the primary cohort. As illustrated in Figure 5, the scores assigned on the points scale could match each level of every variable on the nomogram. Therefore, a total score could be obtained by adding the score from various variables or their levels. Finally, the 36-, 60-, and 120-month cancer-specific survival for each individual patient could be estimated and predicted according to the patient's total score on the nomogram. Of note, in the nomogram, the proportion of extent of disease extension is the largest, while that of treatment is the smallest. These findings revealed that current treatment regimens played a limited role in improving the prognosis of patients with PB, and the extent of tumor extension was still a key factor to the prognosis.

### Validation and Calibration of the Nomogram

**Figure 6:**
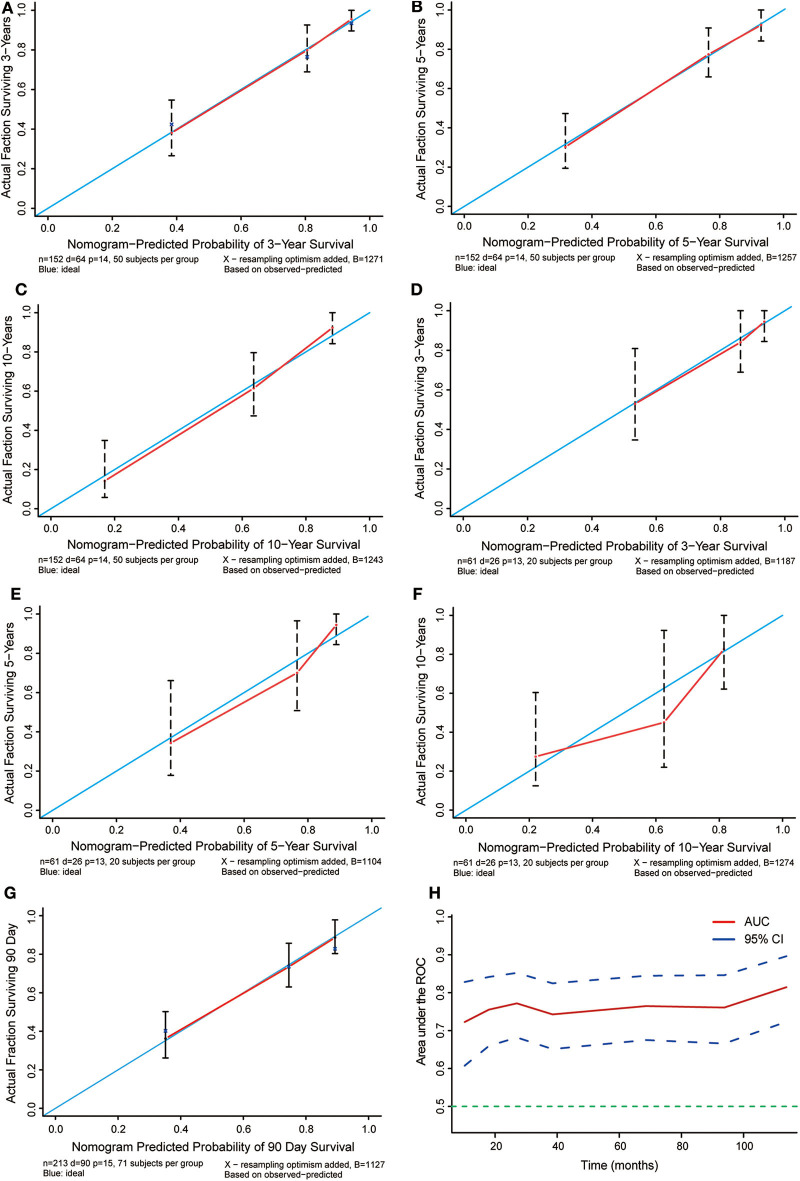
Calibration plots for the prediction of cancer-specific survival at 36-, 60-, and 120-months. **(A–C)** Calibration curves displaying the probability of 36-, 60-, and 120-month cancer-specific survival between the actual observation and the probability predicted by the nomogram in the primary cohort. **(D–F)** Calibration curves showing the probability of 36-,60-, and 120-month cancer-specific survival between the actual observation and the probability predicted by the nomogram in the validation cohort. The blue lines with a slope of 1 were ideal for prediction. **(G)** Cross-validation curves showing the probability of 90 day specific survival between the actual observation and the probability predicted by the nomogram in the validation cohort. The blue lines with a slope of 1 were ideal for prediction. **(H)** Time-dependent ROC curves showing the sensitivity and specificity of the cancer-specific survival prediction by the nomogram.

**Figure 7:**
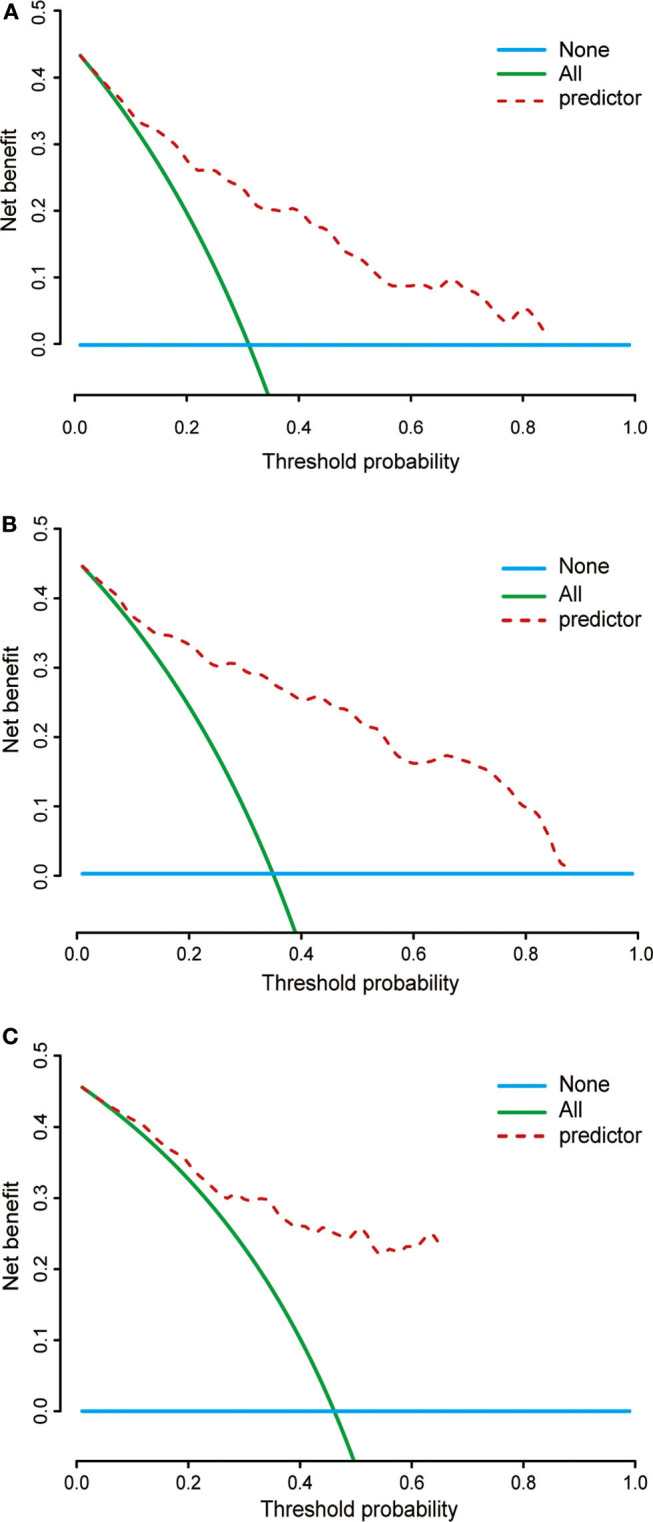
Decision curves of the nomogram predicting 36- **(A)**, 60- **(B)**, and 120-month **(C)** cancer-specific survival. The *x*-axis represents the threshold probability, while the *y*-axis indicates the net benefit calculated by adding the true positives and subtracting the false positives. The horizontal line in parallel with the *x*-axis assumes that cancer-specific death did not occur in any of the patients, while the solid green line assumes that all patients will have cancer-specific death at a specific threshold probability. The brown dashed line represents the net benefit of using the nomogram.

Based on the data of 61 patients in the validation cohort, the C-index of the nomogram for predicting overall survival was 0.802 (95% CI: 0.78–0.83). Additionally, calibration plots of the nomogram showed good consistency between observation and prediction both in the primary (Figures 6A–C) and validation (Figures 6D–F) cohorts for the probability of 36-, 60-, and 120-month survival, as well as the cross-validation in primary and validation cohorts (Figure 6G). Besides, the time-dependent ROC curve was applied to evaluate the discriminative ability of the model. The area under the curve (AUC) value ranging from 0.7 to 0.8 showed the good discriminative ability of this model (Figure 6H). Figure 7 showed the decision curves for the training cohorts to predict the survival probability at 36-, 60-, and 120-month. The blue line represented the net benefit assuming all patients have died, while the green line represented that assuming no patients have died. If the model curve lies in the area between the blue and green lines, it indicates the clinical usefulness of the model. The further away the model curve is from the blue and green lines (that is, the greater the net benefit), the better the clinical value of the nomogram. Of note, great net benefit in the predictive model for almost all of the threshold probabilities at 36- (Figure 7A), 60- (Figure 7B), and 120-month (Figure 7C) was exhibited on the DCA curves. This observation highlighted the potential clinical usefulness of the nomogram.

### Comparison of Predictive Accuracy Between the Nomogram and a Single Independent Factor

The weights of extension and year of diagnosis for survival, shown in Figure 5, were higher than those of other factors. We compared the predictive power for the prognosis of patients with PB between the nomogram, scope of tumor extension, and year of diagnosis. C-indices for the prediction of prognosis by the scope of tumor extension and year of diagnosis were 0.72 and 0.63, respectively. These values were significantly lower than the C-index obtained through the nomogram (0.802; *P* < 0.01).

## Discussion

Identification of risk factors affecting patient survival and the effectiveness of currently applied treatments in rare diseases, such as PB, is of vital importance. Therefore, an abundance of high-quality clinical data is critical to achieving this goal. However, the rarity of PB renders the possibility of conducting prospective clinical trials a difficult task. In this study, the information on 213 adult patients with PB, collected by the SEER database from 1975 to 2016, was retrospectively analyzed. To our knowledge, thus far, this study analyzed the largest series of data regarding PB in adults. The results confirmed the predominant impact of the extent of tumor extension, tumor size, year of diagnosis, age, and different treatment regiments (including RT after surgery, and RT after surgery combined with CT) on the outcome of adult patients with PB. Young patients, especially those not receiving RT during their initial treatment, are often associated with extremely poor outcome (6). Interestingly, the results of this analysis revealed that application of beam RT failed to significantly improve the overall survival of adult patients. In addition, the younger, the more poor prognosis in adolescent and pediatric patients with PB. However, interestingly, our analysis found that there is a diametrically opposite situation in adult patients.

Owing to its rarity, there is limited information with regard to the clinical features and outcomes of adult PB. Moreover, the optimal treatment strategies for adult PB remain to be determined. Our primary target was to identify the clinical risk factors related to prognosis and describe their impact of different risk factors, especially treatment options, on the overall survival of patients. Previously developed nomograms have exhibited higher precision than the conventional staging systems for prognosis in some types of cancers (30, 31). Therefore, we attempted to establish a prognostic nomogram for adult PB based on the clinical data of 213 adult patients with PB evaluate its discriminative ability through a calibration plot and time-dependent ROC curve, and estimate its clinical utility by DCA.

Consistent with the literature (32, 33), the results of this study illustrated that tumor contiguous extension was the most dominant risk factor, and overall survival was greatly reduced in patients with PB for whom tumor extension exceeded the adjacent connective tissues, adjacent organs, or structures and continuously extended. The HR for patients where tumor extension had extended to adjacent connective tissues and adjacent organs or structures vs. those with tumors confined to the gland of origin were 3.70 and 4.74, respectively. This finding indicated that the risk of death in patients with tumor extended to adjacent connective tissues was ~3-fold of that calculated for patients with tumors confined to the gland of origin. Likewise, the HR for patients with further continuous extension vs. those with tumors confined to the gland of origin was 23.31. Interestingly, the similar results were exhibited in the results from the survival analysis of this study. This finding is in line with the results obtained from previous studies concerning metastasis of PB (12, 20). Given that only four patients with further continuous extension were included in this study, with an estimation of 95% CI (4.51–47.68) for the HR, we took into account the conclusions of other studies and consider that the results of this analysis are robust. However, the specific values of these results require further investigation.

As for the effect of tumor size on the prognosis of patients, to date, there is still some controversy in the published literature. Dittmar et al. (34) found that a large tumor size was one of the high-risk factors for mortality. Similarly, a study from Tirada et al. (35) illustrated that increased tumor size was an independent prognostic factor of poor outcome. In addition, Huang et al. (36) demonstrated that the risks of recurrence and death gradually increased when tumor size increased. By contrast, some studies suggested that smaller tumor size is associated with poor survival (37, 38). Consistent with those, the results of this study showed that a larger tumor size was an independent prognostic factor of good outcomes (HR = 0.96, *P* < 0.01), which revealed that for every 1 mm increase in tumor size, the risk of death is reduced by 4%.

Numerous studies demonstrated that the clinical outcome of patients with PB, irrespective of treatment regimens, is worse than that of patients with infratentorial primitive neuro-ectodermal tumors (39–41). It is important to note that surgery remains the first-line therapy in the management of PB, and aggressive surgical resection is recommended (9, 20). In this study, various treatment regimens (i.e., RT, surgical treatment, CT, RT combined with CT, RT after surgery, RT after surgery combined with CT, RT after non-primary surgical procedure combined with CT) were analyzed. The results indicated that these treatment approaches exerted different effects on the prognosis of patients with PB. Consistent with previous studies (42), the results of this study showed that RT after surgery combined with CT was the most dominant therapeutic risk factor, and RT after surgery appears to offer a significant advantage in reducing the risk of death. In detail, the HR for RT after surgery combined with CT vs. beam RT was 0.38, while that for RT after surgery vs. beam RT was 0.47. These findings indicated that the risk of death in patients undergoing RT after surgery combined with CT and those who received the RT after surgery was reduced by 62 and 53%, respectively. Furthermore, the findings were supported by the results of the survival analysis. To date, therapeutic management of adults with PBs is controversial since such few cases exist in the literatures. Multimodality therapy (surgery, RT, and CT) is often attempted and appears to be the optimal approach (43). In the United States, standard treatment of patients with PB currently includes maximal surgical resection followed by adjuvant cranial–spinal irradiation and systemic chemotherapy (43). It was reported that the median progression-free survival rate was 4 months in infants and children under 3 years of age who were treated according to infant brain tumor protocols with intensive chemotherapy alone (33). Surprisingly, Kang et al. (44) in their study found that long-term survival can be achieved for patients who received multimodality treatment, and the results of their study revealed that the median overall survival was 2.3 years, with 2- and 5-year survival rates of 63.6 and 36.4%, respectively, after multimodality treatment. Lee et al. (1) treated 34 adult patients with PB with cranial irradiation therapy and complete surgical resection, and the median survival time was 25.7 months.

Parikh et al. (21) suggested that maximal tumor resection should be the goal for patients aged >5 years with focal disease. Likewise, Mallick et al. (45) showed that patients with subtotal resection and adjuvant RT were often linked to a better survival outcome. In this study, the results of the multivariate analysis could not yet confirm whether surgical treatment alone was the factor affecting prognosis of patients with PB, while survival analysis revealed that the extent of resection is a factor affecting survival of patients with PB. Specifically, comparing to the gross total resection (GTR), RT after GTR, and RT after GTR combined with CT group, survival probability reduced significantly in subtotal resection, RT after subtotal resection, and RT after subtotal resection combined with CT group. In line with our results, another analysis from Tate et al. (20) revealed that a graded increase in survival was observed with increasing degrees of resection (5-year survival rate: 84% for patients who underwent gross total resection vs. 53% for patients who underwent subtotal resection vs. 29% for patients who underwent debulking).

The role of CT in PB remains controversial. According to studies (46, 47), PB appears to be responsive to CT. Nevertheless, the efficacy of CT was not supported by the results of this analysis, which may be related to the scarcity of patients who received CT in this study. Similarly, results from a study revealed that clinical outcomes did not improve after CT in older patients (6). Our analysis also showed that there was no disparity in the average survival time between patients who received CT and those who received RT.

Interestingly, there is also a lack of consensus concerning the effectiveness of RT against PB. Several studies reported that PB was sensitive to RT (40, 48, 49). However, another study supported that PB was radioresistant (50). Based on the results of our analysis, we could not determine whether RT alone was beneficial in the management of PB. In addition, the finiteness of the sample size in the investigation of PB may be the chief cause of this divergence. Consequently, high-caliber clinical trials are urgently warranted to clarify the effectiveness of RT in this setting.

Similar to other studies (51, 52), the results of this analysis revealed that age was a risk factor for the overall survival of patients with PB. Plenty of studies manifested that the younger, the worse the prognosis in pediatric and adolescent patients (20, 21, 53). Nevertheless, a totally opposite trend was presented by the results of this study. In other words, the older, the poorer prognosis in adult patients with PB (HR = 1.15). Addressed concretely, for every 10-year increase in the age of the patient, the risk of death increases by 15%. This may be due to the fact that PB presented the different biological behaviors in adults and juveniles. It is essential to confirm these results in high-quality clinical epidemiological studies involving larger populations.

Several limitations of this study must be acknowledged. First, heterogeneity in the workup, diagnosis, and treatment modalities was unavoidable due to the long period (i.e., >40 years) required to collect the data regarding diagnosis and treatment. Second, data on some critical items reflecting tumor characteristics, such as tumor size and extension, were not available. Although multiple imputations were conducted for missing data during the statistical analysis, the difference between the imputation data and real data was also unavoidable. Third, in the treatment regimens, it was still not known whether the CT regimens were performed before or after surgery. Despite its shortcomings, this analysis has perceptible strengths. Compared with some previous case reports and small-sample analyses, this analysis provided richer information regarding prognostic factors. In addition, inherent biases and heterogeneity were greatly limited, and statistical power was significantly increased. More importantly, the risk factors included in the model are those that are significantly related to PB and markedly easier to acquire than those determined by costly and time-consuming approaches.

In conclusion, five risk factors (i.e., age, year of diagnosis, therapy, extension, and tumor size) were identified in this analysis. Moreover, a nomogram which provided a precise and objective prediction of the prognosis for patients with PB was developed and validated using large-sample data from the SEER database. Importantly, this nomogram is a functional and practical tool that can assist clinicians in evaluating the prognosis of patients and determining appropriate therapeutic strategies. Additional studies are warranted to confirm the clinical value of this model.

## Data Availability Statement

Publicly available datasets were analyzed in this study. This data can be found here: https://seer.cancer.gov/data/.

## Ethics Statement

All authors signed the SEER Research Data Agreement to protect the privacy of the patients, which is consistent with ethical principles.

## Author Contributions

All authors listed have made substantial, direct and intellectual contribution to the work, and approved it for publication.

## Conflict of Interest

The authors declare that the research was conducted in the absence of any commercial or financial relationships that could be construed as a potential conflict of interest.

## Abbreviations

PB: pineoblastoma
C-index: concordance index
ROC: receiver operating characteristic
DCA: decision curve analysis
AUC: area under the curve
PPT: pineal parenchymal tumor
SEER database, Surveillance, Epidemiology: and End Results database
ICD-O-3, International Classification of Diseases for Oncology: third Edition
WHO: World Health Organization
BRT: beam radiotherapy
CT: chemotherapy
GTR: gross total resection
