# Corrigendum: Development and Validation of a Prognostic Nomogram to Predict Cancer-Specific Survival in Adult Patients With Pineoblastoma

**DOI:** 10.3389/fonc.2020.594049

**Published:** 2020-10-02

**Authors:** Yajun Jing, Wenshuai Deng, Huawei Zhang, Yunxia Jiang, Zuoxiang Dong, Fan Fan, Peng Sun

**Affiliations:** ^1^Department of Neurosurgery, Affiliated Hospital of Qingdao University, Qingdao, China; ^2^Department of Pharmacology and Toxicology, University of Mississippi Medical Center, Jackson, MS, United States; ^3^Department of Nursing, Medical College of Qingdao University, Qingdao, China

**Keywords:** pineoblastoma, nomogram, prognosis, risk factor, C-index

In the original article, there were two mistakes in the legends for the ***Kaplan–Meier curves*** and the ***nomogram***as published. The Kaplan-Meier curves in the section ***Independent*** ***Prognostic Factors in the Primary Cohort***were supposed to be **Figure 4**, but were incorrectly included as **Figure 5**. In addition, the nomogram in the section ***Prognostic*** ***Nomogram of Overall Survival***was supposed to be **Figure 5**, but it was incorrectly included as **Figure 4**. The correct legends appear below.

**Figure 4 |** Kaplan–Meier curves for patients with PB according to different independent prognostic factors. The Kaplan–Meier curves for patients with primary PB according to age **(A)**, year of diagnosis **(B)**, tumor size **(C)**, RT after GTR & CT or RT after subtotal resection & CT **(D)**, GTR and subtotal resection **(E)**, RT after GTR or subtotal resection **(F)**, RT, RT after surgery & RT after surgery & CT **(G)**, extent of tumor extension **(H)**.

**Figure 5 |** Nomogram predicting 36-, 60-, and 120-month cancer-specific survival for patients with PB. Prognostic factors including age, race, tumor extension, tumor size, and therapy, and the scores assigned on the points scale could match each level of every variable on the nomogram. Thus, a total score was obtained by adding the score from various variables or their levels. Finally, the 36-, 60-, and 120-month cancer-specific survival for each individual patient could be estimated on the basis of the total score. ^*^p < 0.05, ^*^^*^p < 0.01, ^*^^*^^*^p < 0.001.

In the original article, there was an error. The nomogram referenced in the section ***Comparison of Predictive Accuracy Between the Nomogram and a Single Independent*** ***Factor***was supposed to be **Figure 5**, but it was incorrectly written as **Figure 4**.

A correction has been made to the **Results** section, sub-section ***Comparison of*** ***Predictive Accuracy Between the Nomogram and a Single Independent Factor*,** ***paragraph 1****:*

The weights of extension and year of diagnosis for survival, shown in **Figure 5**, were higher than those of other factors. We compared the predictive power for the prognosis of patients with PB between the nomogram, scope of tumor extension, and year of diagnosis. C-indices for the prediction of prognosis by the scope of tumor extension and year of diagnosis were 0.72 and 0.63, respectively. These values were significantly lower than the C-index obtained through the nomogram (0.802; *P* < 0.01).

The authors apologize for these errors and state that this does not change the scientific conclusions of the article in any way. The original article has been updated.

